# A Treatise on Therapeutics

**Published:** 1876-09

**Authors:** 


A TREATISE ON THERAPEUTICS, COMPRISING MATERIA MEDICA AND TOXICOLOGY, WITH SPECIAL REFERENCE TO THE APPLICATION OF THE PHYSIOLOGICAL ACTION OF DRUGS TO CLINICAL MEDICINE. By H. C. Wood, Jr., M.D., Professor of Botany and Clinical Professor of Diseases of the Nervous System in the Medical Department of the University of Pennsylvania, Physician to the Philadelphia Hospital, etc. Second edition, revised and enlarged. Philadelphia. J. B. Lippincott & Co. 1876.

The fact that the first edition of this work was exhausted in a few months after it was issued, is the greatest evidence of the high estimate in which the work is held by the profession. This, the second edition, is revised and enlarged with great care, embracing a number of subjects not discussed in the first edition, as coffee, tea, tobacco, salicylic acid, picric acid, cold, heat, electricity, and many others. The well-known position of the author, and the high estimation in which he is held by the profession, and above all, the almost unprecedented sale of the first edition, make it evident that this book requires no commendation at our hands.

